# Utilization of Orthodontic Services in the Fars Province, Iran: The Reasons People Travel to the Capital for Orthodontic Treatment

**Published:** 2015-09

**Authors:** Vahid Moshkelgosha, Hamideh Azar, Ali Golkari, Mohammad Reza Azar

**Affiliations:** 1 Dept. of Orthodontics and Orthodontics Research Center, School of Dentistry, Shiraz University of Medical Sciences, Shiraz, Iran.; 2 Students Research Committee, School of Dentistry, International Branch, Shiraz University of Medical Sciences, Shiraz, Iran.; 3 Dept. of Dental Public Health, School of Dentistry, Shiraz University of Medical Sciences, Shiraz, Iran.; 4 Dept. of Endodontics, School of Dentistry, Shiraz University of Medical Sciences, Shiraz, Iran.

**Keywords:** Orthodontics, Dental Health Services, Utilization, Travel

## Abstract

**Statement of the Problem:**

The first step towards appropriately planning orthodontic treatment and prevention services is to understand the needs and demands of the target population. However, this is ignored in most developing countries.

**Purpose:**

This study aims to evaluate the attitudes of patients in Fars province towards travelling to the provincial capital (Shiraz) to receive orthodontic treatments.

**Materials and Method:**

This cross-sectional study randomly selected 420 patients referring to two public and four private orthodontic centers in Shiraz, coming from other regions of Fars province. The patients were interviewed about their demographic status, residing area and the reasons of deciding to receive orthodontic treatment in Shiraz. Data were analyzed by SPSS software, using Chi-square and ANOVA statistical tests.

**Results:**

The response rate was 96% (N=403). Near half of the patients (176; 43.7 %) lived 100-200km, and 80 (19.9%) patients lived farther than 200km from Shiraz. Having no orthodontist (54.8%) and no dentist providing orthodontic treatment (39.2%) in their region were the most important factors bringing the patients to provincial capital for orthodontic treatments. High costs of treatment in their home county and hoping to receive treatment of higher quality were the other important motives for lower and higher socio-economic backgrounds, respectively. Friends'/relatives' recommendation was a more important factor in patients using public clinics for choosing their therapist, while referral from dentists was more important for patients going to private clinics. The patients who lived in counties farther than 200km from Shiraz were more concerned about quality of treatment results than those who lived closer (*p*= 0.010).

**Conclusion:**

A multifactorial approach is needed to provide the desired orthodontic services for people in Fars province so that they would not need to travel to the capital for such treatments.

## Introduction

Orthodontic treatment, in public's viewpoint, is regarded as a way to improve personal appearance, augment oral health, and increase self-confidence. Although recent progresses in preventive dentistry have reduced the need for general dental services, the needs and demands for orthodontic treatments have not declined.[1-3] Not all of these needs and demands could be met due to the lack of recourses.[4-6] Therefore, proper orthodontic services should be planned and implemented to achieve the optimum results.[7-8] Difficulty to access specialists in distant areas is considered as an even more important factor than financial matters when considering the barriers to seek orthodontic services.[9-10]

Fars is a vast province in Iran that holds more than one million individuals in the age range of 5-17 years[11] who are the main target group for orthodontic services. Previous studies on Fars population have shown a relatively high level of needs and demands for orthodontic treatment.[12-13] Unfortunately, the people who reside in most counties do not have access to orthodontic care in their neighborhood due to poor distribution of specialist services.[14]

The decision whether to take orthodontic treatment or not, and the selection of orthodontic center is largely influenced by friends and close relatives.[15] Studies have shown that people decide differently about orthodontic treatment compared with other dental treatments. Patients do more research and look for the best options to choose their orthodontic center. More people intend to travel longer for orthodontic treatment than for other general or specialized dental treatments, especially if the parents have had orthodontic treatment, or desired to have.[16]

Planning proper orthodontic services within a public health system requires information on the orthodontic treatment needs and demands of populations and their attitudes and expectations towards such treatments,[1, 17] and unfortunately, few such studies have been conducted in Fars province.[12-13] This study was designed and conducted to evaluate the reasons of people for traveling to the Fars capital (Shiraz), demanding orthodontic treatment.

## Materials and Method

A cross-sectional study was designed. The study population consisted of patients residing in any part of Fars province except the city of Shiraz who were admitted to a public or private orthodontic clinic of the city of Shiraz. Two public dental clinics and four orthodontic private practices were selected for sampling. A sample size of 380 was calculated appropriate. Assuming a response rate of 90 percent, 420 patients were selected for data acquisition. The patients were selected randomly by their registered number from the current patients' register in each clinic or practice. The number of patients selected from each dental center was calculated separately according to the total number of registered patients in that center. The inclusion criteria were that the patient should have been admitted for comprehensive orthodontic treatment, as well as living in any of the counties of Fars province other than Shiraz.

Selected patients were invited to the study. The purpose and process of the study was explained to them. Those who accepted to take part were interviewed by trained and calibrated dental staff. In case the patient was dependent on their parents in making decisions about treatment options, one of the parents were asked to participate in the interview to help answering the questions. The subjects that were unwilling to cooperate or giving unclear answers to the questions were excluded from the study. 

The participants were asked about their age, education level, employment status, the county they are resident in, and type of transportation they usually use for traveling to and from their orthodontics center. They were also asked about their main reasons for attending the orthodontic centers in Shiraz. Semi-open questions were used. However, the interviewers used clues about different aspects of orthodontic treatment such as accessibility to specialist, cost, infection control, quality of treatment, and referrals to elicit answers from the patients about these issues. 

Statistical Package for Social Science (SPSS version PASW 18) was used for data entry and analysis. Descriptive statistics were used to describe the study population. Pearson’s Chi-Square test was used to assess the differences in answers between participants with different demographic backgrounds. One-way ANOVA was used to compare the answers between persons residing in different counties. The significance level was set at 0.05. Motivating reasons were compared between demographic groups using a Chi-Square test.

## Results

Out of 420 patients selected for the interview, 17 refused to participate or did not cooperate during the interview. Therefore, the information gathered from 403 subjects (response rate= 96%) was used for analysis. Table 1 shows socio-demographic information of the subjects. More than 40% (N=173) of the patients were under 18 years old, for whom their parents were interviewed. The average age of respondents was 30.8±1.3 years. Number of female respondents (248; 61.5%) was markedly higher than males. Among those selected, 292 (72.5%) were attending public and 111 (27.5%) were attending private clinics/practices.

**Table 1:** Distribution (%) of respondents according to socio-demographic, and orthodontic care related variables (N= 403)

**Variable**	**Groups**	**Number** **(%)**
Patients' age	<18 yrs	173 (42.9)
	18-24 yrs	131 (32.5)
	25-29 yrs	64 (15.9)
	>30 yrs	35 (8.7)
Respondents' gender	Woman	248 (61.5)
	Man	155 (38.5)
Respondents' education	Up to high school diploma	227 (56.3)
	University first degree	154 (38.2)
	Post graduate education	22 (5.5)
Employment status	House keeper/unemployed	214 (53.1)
	Employed	189 (46.9)
Person who decides treatment	The patient	230 (57.1)
	Family	173 (42.9)
Type of transportation	Public	221 (54.8)
	Private	182 (45.2)
Type of dental clinic attending	Public	292 (72.5)
	Private	111 (27.5)

**Figure 1:**
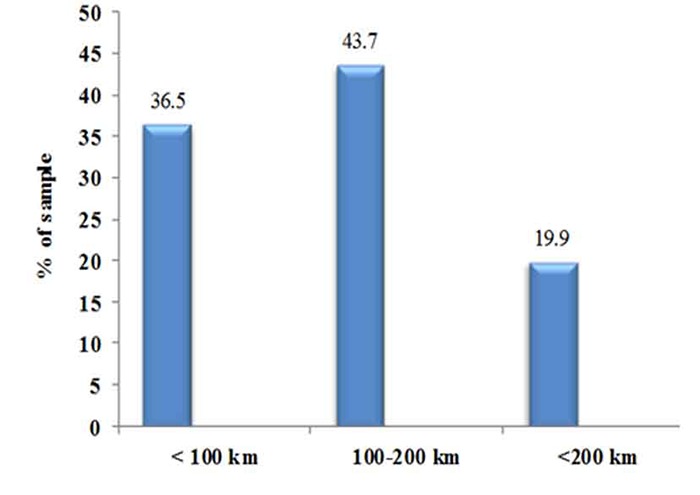
Distribution (%) of patient’s home county distance to Shiraz in kilometers

More than one third of patients (147; 36.5%) lived in counties less than 100 kilometers far from Shiraz (Figure 1).

**Figure 2:**
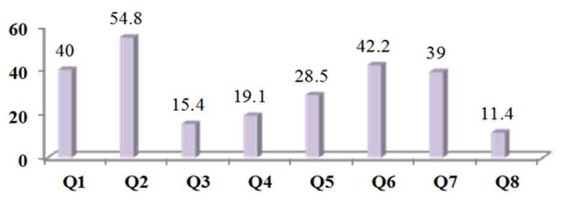
Distribution (%) of reasons for traveling to Shiraz for orthodontic treatment. Patients could cite more than one reason (Q1.lack of orthodontic service provision in home county, Q2.lack of orthodontic specialist in home county, Q3.high cost of treatment in home county, Q4.not sure of proper infection control in home county clinics, Q5.not sure of quality treatment results in home county, Q6.advice of friends or relatives, Q7.refferal from a dentist in home county, Q8.having a second residing place in Shiraz)

The lack of orthodontist in patients' counties was the reason for travelling to Shiraz for orthodontic treatment in more than half of patients (221; 54.8%). About 40% of them (N=158) said no orthodontic services were provided in their hometown even by general dentists. Taking advice from friends and relatives to go to a specific orthodontist and referral to a specific orthodontist by their general dental practitioner were other important factors to choose their clinic in Shiraz. Figure 2 shows the rate of patients' motivating reasons for traveling to Shiraz for orthodontic treatment.

No difference was found between sexes or different age groups in motivating factors mentioned for demanding orthodontic treatment in Shiraz instead of their hometown. The patients with higher education level were more motivated by reasons like access to orthodontic specialist, quality of treatment, and referrals than those with less education (*p*< 0.05). Having no access to orthodontic services (both specialist and non-specialist) was a more important reason for patients using public transport system than those who used private means of transportation (*p*< 0.05). In the latter group, the quality of treatment results was the most important factor for choosing their orthodontist (*p*< 0.05).

Significant differences were found in attitudes between patients attending public and private clinics. Apart from the lack of orthodontic service provision in their hometown, high costs of treatment in their home county was a more important factor for those attending treatment in Shiraz public clinics (42; 19.0%) than those attending private ones (20; 11%) (*p*= 0.027). However, quality of treatment results (35.2% and 23.1%; *p*= 0.008) and infection control issues (23.6% and 15.4%; *p*= 0.036) were more motivating for patients attending private clinics. While taking advices from friends or relatives was more common factor for patients using public clinics (*p*= 0.001), referral from dentists was more important for patients going to private clinics (*p*= 0.043).

The patients who lived in counties farther than 200km from Shiraz were more concerned about quality of treatment results (62 out of 80; 77.5%) than patients who lived in shorter distances (201 out of 323; 62.2%). The difference was statistically significant (*p*= 0.010). No other differences were found associated with the distance the patients had to travel.

## Discussion

Need and demand for orthodontic treatments is relatively high, both for the concentrated urban population of Shiraz[12] and for the people living in smaller towns and rural areas.[13] However, there is unequal distribution of orthodontists in this province. Only a few orthodontists work in other cities of the province apart from Shiraz.[14] Considering the frequent visits and the prolonged nature of orthodontic treatments, travel would be time consuming, costly, and frustrating. On the other hand, considering the main age group of orthodontic patients, many school and working hours would be missed by the patients and/or their companions.

The demand for orthodontic services was higher in women than in men for all counties even though the previous clinical data from Fars province suggest that need may be equal or even greater for men.[12-13] There are reports similar to the findings of the current study in other populations.[18-19]

In this study, in the contrary to unwillingness of people from lower socio-economic background in other countries,[5, 20] patients from both low and high socio-economic backgrounds travel long distances summoning orthodontic treatments as previous studies on Fars population confirm.[13] The difference could reflect the cultural differences.

In this study, the data were collected from subjects who were undergoing orthodontic treatments. Therefore, some patients who need orthodontic treatment but could not seek it due to barriers might have been lost. Further population-based surveys are required in each county to collect supplementary data. The findings of this study can act as a guideline for further investigations before provision of orthodontic workforce and centre in any Fars province county.

## Conclusion

About one- third less patients would have to travel to the provincial capital if orthodontic treatment services were provided in their hometowns. More patients from educated, high socio-economic families would prefer to travel longer distance to go to their desired orthodontist than those from less educated, lower socio-economic families.
